# Phantom of the immunologic opera: Unmasking the role of innate lymphoid cells (ILC) in inborn errors of immunity (IEI)

**DOI:** 10.70962/jhi.20250045

**Published:** 2025-09-10

**Authors:** Ahmet Eken, Sara Johnson, Serife Erdem, Elena W.Y. Hsieh

**Affiliations:** 1 Department of Immunology and Microbiology, School of Medicine, University of Colorado Anschutz Medical Campus, Aurora, CO, USA; 2 Department of Medical Biology, School of Medicine, Erciyes University, Kayseri, Turkiye; 3 Department of Immunology, School of Medicine, Kirsehir Ahi Evran University, Kirsehir, Turkiye; 4 Genome and Stem Cell Center, Kayseri, Turkiye; 5 Department of Pediatrics, Section of Allergy and Immunology, Children’s Hospital Colorado, University of Colorado Anschutz School of Medicine, Aurora, CO, USA

## Abstract

Just as the Phantom orchestrates events from the shadows of the Paris Opera House, innate lymphoid cells (ILC) operate behind the scenes of the immune system, shaping immune responses without the antigen specificity of their T cell counterparts. While more understudied than their better-known T cell counterparts, these enigmatic cells serve as first responders to infection and tissue disruption, playing crucial roles in mucosal immunity and homeostasis—packing an iron-fist punch under a velvet glove. However, in the context of inborn errors of immunity (IEI)—a diverse group of over 500 monogenic disorders affecting immune function—the role of ILC remains largely unmasked. While traditionally overlooked, recent patient studies reveal that ILC dysfunction contributes to disease pathogenesis in at least 19 distinct IEI, raising critical questions: Are ILC indispensable protectors, or do they represent a redundant act within the immune repertoire? How do they respond to standard treatments such as hematopoietic stem cell transplantation (HSCT)? In this review, we unveil the hidden roles of ILC in IEI, analyzing their developmental and functional defects, their role in immune dysregulation, and their therapeutic potential. Much like the Phantom’s elusive presence, ILC may hold the key to understanding immune resilience and designing novel treatments for immunocompromised patients.

## Teaser

This review unmasks the significant, yet understudied, role of innate lymphoid cells in inborn errors of immunity. The authors examine how these “behind the scenes” immune components contribute to monogenetic immune disorders, exploring their developmental defects, role in immune dysregulation, and potential as therapeutic targets.

Just as the Phantom operates unseen beneath the Paris Opera House, shaping the narrative from the shadows, innate lymphoid cells (ILC) work behind the scenes of our immune system, helping to orchestrate critical immune responses without taking center stage like their more famous T cell counterparts. In this review, we unmask these mysterious cellular actors and reveal how their dysfunction can contribute to the pathogenesis in inborn errors of immunity (IEI). Much like uncovering the Phantom’s enigmatic presence through haunting melodies and mysterious backstage events, recent and ongoing investigations are barely beginning to decipher how these elusive cells shape our fundamental understanding of (mostly) local-tissue and peripheral immune responses, particularly when genetic mutations disrupt their normal function.

Our analyses of recent patient studies showing ILC dysregulation in 19 different monogenic IEI suggest that ILC may be an untapped therapeutic target. The review is presented in four acts—Act 1 sets the stage, briefly highlighting the discovery, development, and functions of various ILC subsets; Act 2 describes the linkage between ILC disruption and specific IEI categories (based on the International Union of Immunological Societies [IUIS] classification); Act 3 examines evidence, both for and against, the redundancy of ILC in the immune system; and Act 4 focuses on how expanding knowledge of ILC development, differentiation, and function may be exploited to generate novel therapies for IEI disorders.

## ACT 1: Setting the stage—lymphoid cells that infiltrate, adapt, and respond without antigen specificity

### The lymphoid identity crisis: The discovery of ILC

Natural killer (NK) cells were first identified in 1975 as innate lymphocytes capable of lysing virally infected and tumor cells without prior sensitization (1). For years, NK cells were considered the sole inhabitant of the innate lymphoid population, essential for early responses to intracellular pathogens and tumors via cytotoxicity and interferon (IFN)-γ production. Additional subsets of distinct innate lymphocytes were identified from 1992 onward, including lymphoid tissue inducer (LTi) cells (2, 3, 4) and helper-like innate lymphocytes (5, 6, 7, 8, 9, 10, 11, 12, 13, 14, 15). The term “innate lymphoid cells” was coined in 2013, with classification grouping them into cytotoxic NK cells and noncytotoxic helper-like ILC1, ILC2, and ILC3 subsets based on developmental and functional criteria (16).

**Figure 1.**
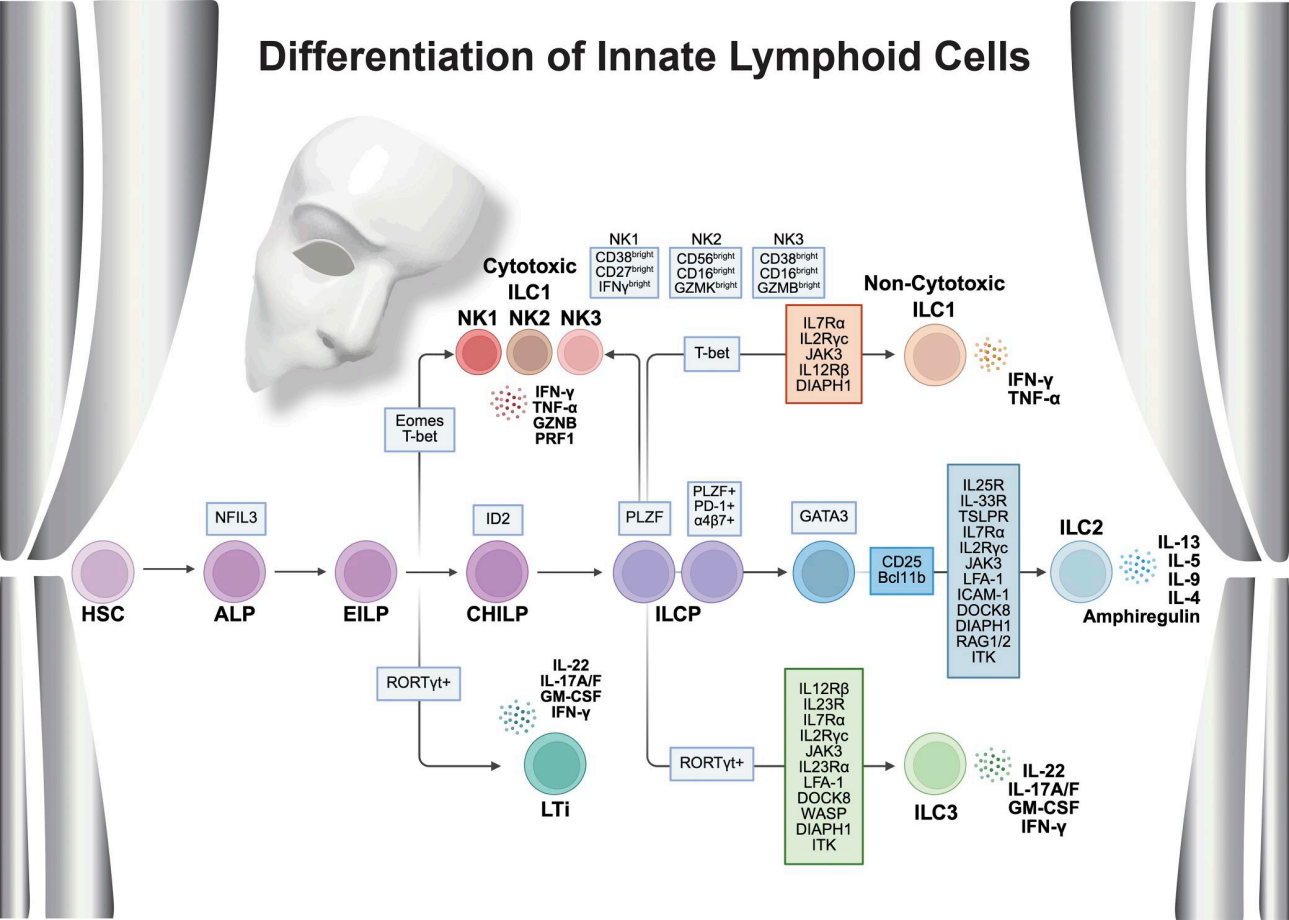
**Critical genes regulating the differentiation and functions of ILC.** HSC, hematopoietic stem cell; GZNB, granzyme B; PRF1, perforin 1; NFIL3, nuclear factor, IL 3 regulated; TOX, thymocyte selection-associated high-mobility group box; TCF1, T cell factor 1; PLZF1, promyelocytic leukemia zinc finger; PD-1, programmed death; ICOS, inducible T cell co-stimulator; Rorα, RAR-related orphan receptor α; ICAM-1, intercellular adhesion molecule 1; RAG, recombination-activating genes; Rα, receptor α; Rβ, receptor β; Eomes, eomesodermin.

All lymphoid progenitors (ALP), which include α4β7^+^ and α4β7^−^ common lymphoid progenitors (CLP) in the fetal liver and bone marrow, serve as a shared developmental foundation for both adaptive lymphocytes (T and B cells) and ILC. The transition from CLP to ILC precursor (ILCP) depends on specific transcription factors (TF) such as T cell factor 1 (17), inhibitor of DNA binding 2 (ID2) (18), and promyelocytic leukemia zinc finger (PLZF) protein. Within ILCP, these TF establish the programming necessary for development of distinct subsets (19) (Fig. 1). Intermediate progenitors with distinct phenotypic features have been defined between ALP and ILCP, including early innate lymphoid progenitors (EILP) and common helper innate lymphoid progenitors (CHILP). Furthermore, recent studies have revealed NK cells can develop from both pre-ILCP (PLZF−) and ILCP progenitors (PLZF+), with each pathway exhibiting distinct transcriptional profiles (20, 21). Similarly, the development of non-NK ILC subsets also relies on carefully orchestrated transcriptional control from the ILCP stage. Tightly regulated transcriptional programing orchestrates the emergence of ILC1, ILC2, and ILC3 from ILCP and maintains the delicate balance of innate immune responses in mucosal barriers and other tissues (22). Epigenetic modifications further fine-tune this process, creating stable cellular identities and preserving the plasticity needed to respond to changing environmental signals and inflammatory conditions (23, 24).

The discovery of ILC radically expanded our understanding of innate immunity by revealing previously unknown cell populations that function in immune regulation, mucosal defense, and tissue homeostasis. Unlike T and B cells, ILC lack rearranged antigen receptors, yet mirror adaptive T helper (Th) cell functions. They respond rapidly to cytokines and microbial patterns, providing protection during the early stages of infection before the adaptive immune system becomes fully activated.

### Friend or foe: ILC in health and disease

#### Friend: The role of ILC in adaptive immunity, host defense, and tissue homeostasis

ILC play a significant role in early immune responses and serve as a bridge between innate and adaptive immune responses (23, 25, 26, 27, 28, 29, 30, 31). Evidence from multiple mouse studies (20, 23, 25, 26, 27, 28, 29, 31) and emerging human data demonstrates that ILC (32) actively shape adaptive immune responses through both direct and indirect mechanisms (23). In certain conditions, ILC2 and ILC3 express major histocompatibility complex (MHC) class II molecules, allowing them to present antigens directly to T cells (23, 25, 26, 27, 28, 29, 30, 31). Beyond this antigen-presenting capacity, ILC establish direct interaction and crosstalk with adaptive immune cells through context-specific co-stimulatory molecule expression, including CD80, CD86, OX40 ligand, and inducible T cell co-stimulator ligand, among others (23, 33, 34, 35, 36, 37). Additionally, ILC-derived cytokines promote differentiation and commitment of their Th cell counterparts during the early stages of an immune response and later influence Th function. For example, ILC1-derived IFN-γ promotes T helper 1 (Th1) development, ILC2-produced interleukin (IL)-4 supports Th2 polarization, and ILC3-secreted IL-17 and IL-22 enhance Th17 responses (23, 38, 39). Furthermore, ILC may modulate humoral responses via their secretion of IL-5 and IL-6, which support B1 cell self-renewal and enhance IgM and IgA production (23, 40, 41, 42). ILC further promote adaptive immunity by creating microenvironments conducive to adaptive immune cell function. This effect is largely mediated by ILC influence on important bystander cells. For instance, LTi cells play an essential role in the development and organization of lymph nodes and Peyer’s patches through their influence on stromal cells via lymphotoxin production. In other contexts, ILC promote cell migration, orchestrating the arrival of the right cells to the right location at the right time. This multifaceted interaction between ILC and adaptive immune cells ultimately ensures coordinated immune responses tailored to specific pathogenic challenges.

The innate role of ILC in host defense is largely mediated through their rapid production of specific cytokines. In general terms, this means ILC1 defend against type I pathogens via IFN-γ and tumor necrosis factor (TNF)-α production (43, 44, 45, 46), ILC2 defend against type II pathogens via IL-4, IL-5, and IL-13 production (40, 47), and ILC3 defend against type III pathogens via IL-17, IL-22, and granulocyte-macrophage colony-stimulating factor (GM-CSF) production (12, 48, 49, 50, 51, 52, 53, 54, 55, 56). For instance, ILC2 play pivotal roles in defending against helminths and rhinoviruses. They crucially recruit, activate, and support eosinophil survival at inflammation sites primarily through their production of IL-5. Mouse models of *Nippostrongylus brasiliensis* and *Strongyloides venezuelensis* infection demonstrate ILC2 expansion and activation in the lungs, with IL-33 playing a central role in this process (57, 58), while during rhinovirus infection, ILC2 expand in an IL-25–dependent manner (59).

Finally, ILC are tissue-resident cells (5, 10, 60, 61, 62, 63, 64) that can maintain tissue homeostasis, particularly at mucosal surfaces (12, 27, 63, 65, 66, 67, 68, 69). They provide resistance to mucosal inflammation by supporting antimicrobial peptide production, mucus secretion, IgA synthesis, and epithelial regeneration, functions important for protection against infections and preserving barrier integrity, particularly in immunocompromised patients. For more details, see the 2020 review from the Kronenberg Lab (70).

#### Foe: The role of ILC in inflammation, allergy, and autoimmunity/inflammation

Building upon our understanding of ILC and their beneficial roles in both adaptive and innate immunity, we now turn to their darker side. While the previous section explored how ILC provide multifaceted immune protection and maintain tissue homeostasis, this section examines how these same cells can become destructive forces when their activities become dysregulated (45).

ILC1 have been implicated in various inflammatory diseases, with their enrichment observed in the synovial fluid of psoriatic arthritis patients (71) and the intestines of inflammatory bowel disease (IBD) patients (5). Further, this ILC1 enrichment often stems from ILC3 that undergo phenotypic conversion, losing retinoic acid receptor-related orphan receptor γt (RORγt) expression and becoming significant producers of the proinflammatory cytokine IFN-γ (5, 72, 73, 74, 75). This cellular plasticity—the ability of ILC to “switch” from one subtype to another—may exacerbate intestinal inflammation, as seen in both human patients and murine models of IBD (5, 76, 77).

In inflammatory diseases like asthma and atopic dermatitis (AD), ILC2 significantly contribute to pathogenesis. Patients with AD exhibit increased ILC2 numbers, with mouse studies showing ILC2 can induce AD symptoms in T cell-deficient Rag^−/−^ mice (78, 79). These findings underscore the potential of ILC2 to exacerbate atopy in IEI patients. In support of this idea, mouse models where asthma is induced by either house dust mite or papain, Wolterink et al. (80) demonstrated that ILC2 drive allergic airway inflammation via IL-4, IL-5, and IL-13 cytokine production. Conversely, antibodies that target and eliminate ILC2 and other Th2-associated cells in humanized mouse models ameliorate airway inflammation (47).

ILC3 have been extensively investigated in inflammatory diseases, including IBD, psoriasis, rheumatoid arthritis (RA), multiple sclerosis (MS), and ankylosing spondylitis (AS) (45, 81, 82). Importantly, in IBD, IL-23 receptor (IL-23R)–responsive ILC3 are specifically increased in intestinal tissue but reduced in blood, possibly indicating a migration pattern that could serve as a diagnostic indicator for disease activity or therapeutic target (5, 83, 84). Notably, some murine models of IBD also reveal pathogenic roles for ILC3 (55, 74, 85, 86, 87). Increased ILC3 frequency and altered cytokine profiles are observed in psoriatic skin lesions and the joints of RA patients, suggesting their involvement in chronic inflammation and tissue damage (88, 89, 90, 91). While in MS and AS, dysregulated ILC3 responses are linked to neuroinflammation and enthesitis, respectively, highlighting their role in autoimmune processes beyond mucosal tissues (26, 43, 92, 93, 94, 95, 96).

The pathological roles of ILC in inflammatory, allergic, and autoimmune conditions demonstrate their dual nature within the immune system. Like the Phantom lurking beneath the opera house, these cells can shift from protective guardians to destructive forces when their delicate balance is disrupted. The same effector functions that protect against infection and maintain tissue homeostasis can, when dysregulated, initiate pathological processes from the shadows. ILC plasticity—particularly the conversion between subtypes—emerges as a factor in disease pathogenesis, much like the Phantom’s ability to appear and influence events from unexpected locations. Understanding these mechanisms could lead to novel therapeutic strategies targeting specific ILC populations or their plasticity in various inflammatory and autoimmune conditions.

## ACT 2: Behind the mask—characterizing defects in ontogeny, phenotype, and function of ILC in IEI

ILC are functionally parallel to T cell subsets, acting as innate counterparts that respond rapidly during early phases of infection, tissue damage, or homeostatic disruption (10, 15, 16, 56, 97, 98). These early responders provide a powerful first-line defense, bridging the gap until the adaptive immune system is activated (10, 15, 16, 56, 97, 98). However, their importance extends beyond the initial response to pathogens, as ILC continue to contribute dynamically to inflammatory/immune responses as they evolve in both time and tissues.

In the context of IEI, the role of ILC is nuanced—particularly when the immune defect impairs T cell subsets, such as in severe combined immunodeficiency (SCID), combined immunodeficiencies (CIDs), or other T cell-specific defects. On one side, akin to the Phantom’s undamaged face, healthy ILC responses can serve as compensatory mechanisms that mitigate the absence or dysfunction of adaptive T cells in immunocompromised hosts. These ILC help maintain immune competence by controlling infections and preserving tissue homeostasis (10, 12, 16, 27, 56, 97, 99). On the opposite side, akin to the Phantom’s burned face hidden under the mask, aberrant ILC responses contribute to the pathology of immunodeficiency. Support for the latter idea—that disruption of ILC function exacerbates disease—comes from the study of Rag-deficient mice. Pathogenic variants, when combined with recombination-activating genes 1 and 2 (RAG1/2) deficiency, may directly or indirectly impact ILC subsets, leading to conditions such as intestinal inflammation (ILC3), atopy or skin disorders (ILC2), or even tumorigenesis (ILC1) (55, 56, 85, 87). Notably, studies using *Rag2*^*−/−*^*Il2rg*^*−/−*^ mice, which lack T, B, NK cells, and ILC, have been instrumental in understanding the role of ILC in immune responses and are used in almost all ILC studies as negative controls or as hosts for adoptive transfer of ILC (66, 100).

Standard therapies for patients with IEI include antibiotic prophylaxis and/or treatment; immunoglobulin replacement therapy; immunomodulation with small molecule inhibitors, metabolic inhibitors, monoclonal antibodies, small molecule inhibitors and others; hematopoietic stem cell transplantation (HSCT); or gene therapy. These treatments can significantly influence ILC function, interaction with microbiota, and host protective immunity (101). Understanding the extent of ILC dysregulation associated with a particular IEI, how these therapies impact ILC subsets, and their restoration post-HSCT is critical to optimizing treatment outcomes and options.

Despite the fact that >500 distinct IEI have been identified (102), ILC have been studied in only 19 of these IEI (Tables 1 and 2). This striking knowledge gap presents both a challenge and an opportunity for the field. Each newly characterized IEI offers valuable insights into gene-specific roles in ILC development and function. Thus, systematic investigation of ILC across the spectrum of IEI will not only advance our fundamental understanding of innate immunity but also potentially reveal novel therapeutic targets and personalized treatment approaches for these challenging disorders.

**Table 1.** IEI with described ILC abnormalities: Clinical characteristics

Disease	Gene	Inheritance	OMIM	IUIS tables major category; subcategory	Microbial susceptibility	Immune dysregulation
gc deficiency SCID	*IL2RG*	XL	308380	T1; ST1	Recurrent severe viral, bacterial, and fungal infections; diarrhea	Treg defects; hypomorphic mutations in IL-2Rγ result in CID with immune dysregulation
JAK3 deficiency SCID	*JAK3*	AR	600173	T1; ST1	Recurrent or severe respiratory infections; oral thrush (candidiasis); pneumonia	Treg defects
IL7Ra deficiency SCID	*IL7R*	AR	146661	T1; ST1	Recurrent severe viral, bacterial, and fungal infections; candidiasis; chronic diarrhea; *Pneumocystis jirovecii* pneumonia	Treg defects; may present with immune dysregulation; chronic inflammatory diseases
RAG1 deficiency SCID	*RAG1*	AR	179615	T1; ST2	Recurrent severe viral, bacterial, and fungal infections; *Pneumocystis jirovecii*; neutralizing antitype I IFN antibodies associated with varicella infection	Hypomorphic mutations associated with immune dysregulation
RAG2 deficiency SCID	*RAG2*	AR	179616	T1; ST2	Recurrent severe viral, bacterial, and fungal infections; *Pneumocystis jirovecii*; neutralizing antitype I IFN antibodies associated with varicella infection	Hypomorphic mutations associated with immune dysregulation
RORgt deficiency	*RORC*	AR	616622	T6; ST1	Mycobacteria and *Candida albicans*	No broad infectious or autoimmune phenotype
IL-12Rb2 deficiency	*IL12RB2*	AR	601642	T6; ST1	Mycobacteria and *Salmonella*	Deficiency is associated with autoimmunity in mice; human data are scarce
IL-23R deficiency	*IL23R*	AR	607562	T6; ST1	Mycobacteria and *Salmonella*	SNPs associated with autoimmunity, not null or LOF
IL-12R and IL-23Rb1 deficiency	*IL12RB1*	AR	601604	T6; ST1	Mycobacteria and *Salmonella*	Only 3 out of 300 LOF showed autoimmunity; SNPs associated with autoimmunity
LAD1	*ITGB2*	AR	600065	T5; ST2	Recurrent bacterial and fungal infections	Leukocytosis
DOCK8 deficiency (HIES)	*DOCK8*	AR	243700	T1; ST3	Recurrent cutaneous viral, fungal, and staphylococcal infections	Treg defects; low NK cells with poor function; eosinophilia; severe atopy; cancer diathesis
WAS LOF	*WAS*	XL	300392	T2; ST1	Recurrent viral and bacterial infections	Thrombocytopenia with small platelets; eczema; lymphoma; autoimmune disease; IgA nephropathy; vasculitis; XL thrombocytopenia is a mild form of WAS—bloody diarrhea; Treg defects
ITK deficiency	*ITK*	AR	186973	T1; ST3	Epstein–Barr virus (EBV)	EBV-associated B cell lymphoproliferation; lymphoma
BCL11B deficiency	*BCL11B*	AD	617237	T2; ST9	Upper respiratory infections	Immunological dysregulation expressed as allergy, asthma, eczema, eosinophilia, and severe atopy
DIAPH1 deficiency	*DIAPH1*	AR	602121	T2; ST9	Recurrent viral and bacterial infections	B lymphoma; decreased T cell activation/proliferation in vitro; impaired adhesion/microtubule organizing center (MTOC repositioning to immune synapse; defective cytoskeletal organization; mitochondrial dysfunction in SCBMS pathogenesis
STAT3 LOF (HIES)	*STAT3* (*LOF*, *DN*)	AD	102582	T2; ST5	Bacterial infections—boils and pulmonary abscesses, pneumatoceles due to *Staphylococcusaureus*; Pulmonary aspergillus; *Pneumocystis jirovecii*; mucocutaneous candidiasis	Eczema
BTK deficiency (XLA)	*BTK*	XL	300300	T3; ST1	Severe bacterial infections	Normal numbers of pro-B cells
CVID, no gene defect specified	Unknown	Variable	607594 616576 615577 240500 614700 604558	T3; ST2	Recurrent infections	Some show polyclonal lymphoproliferation, autoimmune cytopenias, and/or granulomatous disease

XL, X-linked; AD, autosomal dominant; AR, autosomal recessive; T, table; ST, sub-table; SCBMS, seizures, cortical blindness, microcephaly syndrome.

**Table 2.** IEI with described ILC abnormalities: Peripheral and tissue immune cellular profiles

Disease	Blood total ILC	Blood ILC1	Blood ILC2	Blood ILC3	Tissue ILC	Lymphoid organogenesis	Ig	T cells	B cells	NK Cells	Neutrophils	ILC reference
gc deficiency SCID	Absent	Absent	Absent	Absent	Absent	Absent lymph nodes, tonsils	Low	Very low	Normal to high	Low	Normal	(103)
JAK3 deficiency SCID	Reduced	Reduced	Reduced	Reduced	Reduced	Absent lymph nodes, tonsils	Low	Very low	Normal to high	Low	Normal	(103)
IL7Ra deficiency SCID	Reduced	Reduced	Reduced	Reduced	Reduced	NR	Low	Very low	Normal to high	Normal	Normal	(103)
RAG1 deficiency SCID	Normal to high	Normal to high	Normal to high	Normal to high	Elevated ILC2 in OS	Present	Low	Very low	Very low	Normal to high	Normal	(104)
RAG2 deficiency SCID	Normal to high	Normal to high	Normal to high	Normal to high	Elevated ILC2 in OS	Present	Low	Very low	Very low	Normal to high	Normal	(104)
RORgt deficiency	Not Calculated	Reduced	Increased	Reduced	NT	Absence of palpable axillary and cervical lymph nodes (despite visible tonsils), reduced thymus size	NR	Mild CD4^+^ and CD8^+^ αβ T cell lymphopenia	Normal	Normal	NR	(105)
IL-12Rb2 deficiency	Normal	NT	NT	NT	NT	NR	Normal	Normal	Normal	Reduced cell numbers and function	Normal	(106)
IL-23R deficiency	Normal	NT	NT	NT	NT	NR	Normal	Normal	Normal	Normal numbers; reduced INF-g production	Normal	(106)
IL-12R and IL-23Rb1 deficiency	Normal	NT	NT	NT	NT	NR	Normal	Normal	Normal	Reduced cell numbers and function	Normal	(106)
LAD1	Normal	Normal	Normal	Increased	NT	Present	Normal	Normal numbers, functional defects	Normal numbers	Normal numbers; marginal functional impairment	High in blood	(107)
DOCK8 deficiency (HIES)	Normal	Normal	Reduced	Reduced	NT	NR	Low IgM, normal to high IgG and IgA, high IgE	Low, poor proliferation; few, poorly functioning Treg	Very low CD27^+^ memory B cells; poor peripheral B cell tolerance	Low	Normal counts and burst; some migration and NETosis defects	(108)
WAS LOF	NT	NT	NT	NT	Reduced ILC3	Present, but altered T and B cell distribution	High IgE, low IgM	Progressive decrease in numbers; abnormal lymphocyte responses to anti-CD3	Normal numbers	Normal to high numbers; impaired cytotoxic functions	Normal/high numbers; impaired function	(109, *Preprint*)
ITK deficiency	Normal	Normal	Reduced	Reduced	NT	Present	Variable	Progressive decrease	Normal	Impaired Fc-mediated cytotoxicity	Normal	(108)
BCL11B deficiency	Normal	Normal	Reduced	Normal	NT	NR	Normal	Low	Normal	Subset and maturation defects	NR	(110)
DIAPH1 deficiency	Reduced	Reduced	Reduced	Reduced	NT	NR	Low IgM, normal IgG, variable IgA Near normal vaccine response	Decreased naive T cells and RTEs	High Naive/transitional; low memory	Functional defects	Normal counts and burst; migration and NETosis NT	(111)
STAT3 LOF (HIES)	Normal	Normal	Normal	Normal	NT	Present	High IgE	Normal overall; Th-17 and T-follicular helper cells decreased	Normal; reduced switched and non-switched memory B cells; BAFF expression increased	Variable, both normal and reduced reported	Normal	(108)
BTK deficiency (XLA)	Normal	Normal	Increase Trend	Normal	NT	Underdeveloped lymphoid tissues, including the spleen, lymph nodes, tonsils, and Peyer’s patches	Very low	Normal	Very low	Normal	Variable	(112)
CVID, no gene defect specified	Normal or increased	Normal	Reduced or NT	Increased	NT or ILC3 increased	Variable	Low IgG and IgA and/or IgM	Variable	Variable	Variable	Variable	(112)

NT, not tested; NR, not reported.

### IEI associated with classical or leaky SCID

SCID is defined by the lack (or very decreased) of functional T cells with or without B and NK cell defects (113). According to the latest IUIS update (102), at least 22 single gene defects are associated with SCID, yet only a handful of studies have examined the impact of SCID-associated monogenic defects on ILC ontogeny, phenotype, and function in patients or mouse models with homologous mutations.

#### Common gamma chain (IL2RG) deficiency

The common gamma (γc) chain, encoded by the X-linked *IL2RG* gene, is a crucial component of cytokine receptors for ILs IL-2, IL-4, IL-7, IL-9, IL-15, and IL-21. These cytokines are essential for the development, differentiation, and function of various immune cells, including ILC. Development of all ILC subsets relies on IL-7–mediated signaling. IL-15 signaling is necessary for both NK and helper ILC1 subset development (114). Complete deficiencies in the γc chain result in X-linked SCID in humans, characterized by the absence and/or dysfunction of not only T cells and B cells but also NK cells and all ILC subsets (103). X-linked SCID manifests clinically with increased susceptibility to infections and impaired immune responses.

Mice lacking the γc chain (*IL2Rgc*^*−/−*^*)* exhibit severe immunodeficiency, mirroring human SCID. These mice lack T and B cells, unlike humans who have absent T cells and normal to increased numbers of dysfunctional B cells and have deficiencies in the development and function of NK and helper ILC subsets (LTi, ILC1, ILC2, and ILC3) (74). Absence of functional γc chain impairs signaling through γc-dependent cytokine receptors, particularly IL-7R and IL-15R, leading to defective Janus kinase (JAK) 3 (JAK3)–mediated signal transduction. Similarly, certain hypomorphic mutations of IL2Rγc lead to immune dysregulation in humans (43, 115, 116, 117, 118, 119). Critical questions remain regarding how such mutations influence ILC subset distribution, alter transcriptional and proteomic profiles, and potentially contribute to mucosal inflammation in affected patients. Addressing these questions requires further investigation, using both human tissue and mouse models carrying homologous genetic variants.

#### JAK3 deficiency

JAK3 is a non-receptor tyrosine kinase that belongs to the JAK family, which includes JAK1, JAK2, and tyrosine kinase 2. JAK3 is exclusively associated with the γc chain (120, 121). JAK3 loss-of-function (LOF) mutations result in autosomal recessive form of SCID that lacks T cells but retains B and NK cells. Further, the absence of functional JAK3 disrupts IL-7 and IL-15 signaling, both essential for normal ILC differentiation and survival in humans and mice (103, 122). The cited studies illustrate how investigation of JAK3-deficient SCID has provided crucial insights into normal ILC biology, confirming JAK3’s essential role in their development and function.

JAK3 deficiency in mice blocks ILC differentiation in the bone marrow at the level of the CHILP, resulting in near complete loss of all ILC, including helper ILC subsets and NK cells (122). These findings underscore JAK3’s essential role in ILC development from progenitors, aligning with human studies and highlighting conserved mechanisms across species. The complete absence of ILC in JAK3-deficient mice leads to severe defects in mucosal immunity, including impaired production of IL-5, IL-13, IL-17, and IL-22, cytokines critical for maintaining epithelial barrier integrity and coordinating the immune response to infection.

Patients with JAK3 deficiency exhibit profound reductions in circulating ILC populations, including ILC1, ILC2, and ILC3, as well as diminished tissue-resident ILC in mucosal sites such as the gut and lungs. This widespread ILC depletion would typically increase susceptibility to infection and impair mucosal immunity. Interestingly, despite these significant reductions in ILC numbers, JAK3-deficient patients maintain some immune functions. This suggests that redundant pathways or compensatory mechanisms are at work (103) or, perhaps, that the small number of ILC retained in the tissues are sufficient to protect these specialized immunological niches.

As with other SCID genotypes/phenotypes, patients with hypomorphic JAK3 mutations may present with immune dysregulation, oligoclonal T cell expansion, and organ infiltration and damage, all characteristic of Omenn syndrome (OS) (123, 124). The impact of hypomorphic JAK3 mutations on ILC expansion and pathogenicity remains unclear. For example, it is unknown whether such variant skews ILC populations toward ILC2 as seen in *RAG1/2* hypomorphs in humans (104). These questions require further investigation. In summary, studies in mice and humans demonstrate that JAK3 is indispensable for normal development of ILC via its role in cytokine signaling.

#### IL-7Rα deficiency

IL-7Rα (CD127) forms different heterodimeric cytokine receptors through distinct binding partnerships—pairing with the γc chain (CD132) for the IL-7R and with thymic stromal lymphopoietin (TSLP) receptor (TSLPR) (114, 125). Each receptor subunit interacts with specific JAKs: IL-7Rα with JAK1, γc with JAK3, and TSLPR with JAK2. While both IL-7 and TSLP signaling pathways primarily activate signal transducer and activator of transcription (STAT) 5, IL-7–mediated signaling also activates signal transducer and activator of transcription 3 (STAT3) and STAT1 to a lesser extent. Further, IL-7R STAT5-mediated signaling activates the TF nuclear factor, IL 3 regulated and GATA-binding protein 3 (GATA3) (Fig. 1), which are essential to ILCP differentiation (126, 127).

IL-7Rα-deficient mice exhibit significant reductions across all ILC subsets, particularly ILC2 and ILC3. Some ILC1/NK cells still develop in these mice, likely due to compensatory IL-15R signaling (128). IL-7 knockout (KO) mice (IL-7^−/−^) show similar, but less severe, reductions in ILC, with ILC2 and ILC3 development again most impaired (128). Importantly, IL-7^−/−^ mice supplemented with recombinant FMS-like tyrosine kinase 3 ligand (Flt3L) modestly increase both ILC2 and ILC3 numbers. This effect was shown to be mediated by Flt3L acting on ILCPs, which express Ftl3 (CD135); while exposure of sorted, terminally differentiated ILC to Flt3L did not improve ILC expansion or survival (129). Finally, studies conducted in mouse models reveal that the IL-7/STAT5 axis also regulates ILC survival by upregulating cellular Fas associated death domain-like interleukin 1β converting enzyme (FLICE)-like inhibitory protein, an anti-apoptotic molecule that acts as a master regulator of the extrinsic apoptosis pathway (130).

In humans, IL-7Rα deficiency causes a form of SCID characterized by a lack of T cells, but normal numbers of B and NK cells. From the ligand perspective, IL-7 cytokine deficiency is very rare, and the extent of lymphocyte defects are unknown, although a similar phenotype would be expected. The importance of IL-7 receptor signaling for human ILC development was established by Vely et al. (103) Their landmark study showed SCID patients with IL2Rγc or JAK3 mutations had reduced numbers of all ILC subsets in blood and tissues. Notably, ILC restoration occurred only in patients treated with a myeloablative regimen prior to HSCT —findings corroborated in comparably treated IL-2Rγc–deficient mice (103). Ex vivo studies by Kyoizumi et al. (131) demonstrated that Notch signaling in combination with IL-7 induced ILC3 commitment and differentiation from human EILP. To date, no studies report investigation of ILC ontogeny, phenotype, and function in IL-7–, IL-7R–, or JAK1-deficient patients. Our own studies of dedicator of cytokinesis 8 (DOCK8)–deficient patients revealed a stepwise pathological cascade—reduced IL-7Rα expression led to diminished IL-7–mediated STAT5 activation, which subsequently resulted in decreased ILC numbers and compromised patient survival (132).

Together, these findings highlight the role of the IL-7/IL-7R signaling axis in both ILC development and maintenance. Disruptions in this pathway whether at the receptor, ligand, or downstream signaling level consistently result in compromised ILC populations across multiple lineages with significant consequences for immune function and host defense.

#### RAG1/RAG2 deficiencies

RAG1 and RAG2 are essential for V(D)J recombination, the process that generates antigen receptor diversity in both T and B lymphocytes. The exact function of RAG expression in ILC development remains an active area of research. It appears to influence cell fate decisions and possibly epigenetic programming during ILC development, even though mature ILC do not require RAG for their effector functions (133, *Preprint*). Although adaptive immunity is severely compromised in RAG deficiency, innate immune cells, including ILC, may remain present (103), though likely with functional impairments (133, *Preprint*).

Although ILC develop independently of RAG-mediated recombination, multiple studies have shown that ILC numbers are elevated in *Rag1*^*−/−*^ and *Rag2*^−/−^ mice, distinguishing their developmental requirements from those of conventional T and B lymphocytes (10, 74, 85, 134). More recent findings suggest that RAG deficiency in mice leads to the specific expansion and activation of ILC2 at steady state, and further, that increased production of IL-5 and -13 by these cells exacerbates inflammation in AD-like disease (133, *Preprint*).

In humans, biallelic LOF mutations in *RAG1* or *RAG2* typically result in SCID, characterized by the absence of T and B lymphocytes (135), while hypomorphic mutations result in OS, leaky SCID (LS), or delayed CID. Among these phenotypic manifestations of RAG deficiency, only OS patients with hypomorphic variants show increased ILC2 frequency along with heightened Th2 response (104). Patients with RAG mutations often present with immune dysregulation, manifesting as autoimmunity or hyperinflammatory disease, indicating potential alterations in innate immune cell function (135). Although complete LOF mutations of RAG1/RAG2 did not alter ILC frequency in humans (104), the exact impact of RAG deficiencies on human ILC subsets requires further investigation beyond simple phenotyping and number analyses.

#### IL-2–inducible T cell kinase deficiency

IL-2–inducible T cell kinase (ITK), a key player in T cell receptor signaling, is essential for the development and function of Th2 cells and impacts the production of Th2 cytokines (IL-4, IL-5, and IL-13) (136, 137). Research using ITK-deficient mice revealed that ITK is required for ILC2 survival and maintenance in the intestines, but not other tissues, and to promote tissue barrier integrity (138), highlighting the essential role of ITK in maintaining ILC2 homeostasis and function.

In 2019, Eken et al. studied a human patient with a novel ITK mutation (108). Ex vivo analyses revealed various T cell defects, including impaired proliferative response, decreased production of Th17-associated cytokines (IL-17A, IL-22, and GM-CSF), and increased Th1-associated IFN-γ production. Additionally, the patient exhibited reduced numbers of ILC2 and ILC3. ITK deficiency in humans typically presents as hypogammaglobulinemia and CD4^+^ T cell loss, often with EBV-associated B cell lymphoproliferative syndrome. It is unclear to what extent loss of ILC in ITK deficiency contributes to these pathologies.

Collectively, these studies underscore the importance of ITK in the development and function of ILC, with deficiencies leading to notable alterations in ILC homeostasis in both mice and humans.

#### B cell lymphoma/leukemia 11B (Bcl11b) deficiency

Bcl11b is a zinc finger TF expressed by both T cells and ILC2 (139, 140, 141, 142, 143, 144, 145). It is expressed at multiple T cell developmental stages, including the DN2 to DN4 transition, DP thymocytes, and mature CD4^+^ and CD8 T+ cells. In pro T cells, Bcl11b critically suppresses differentiation into NK and ILC lineages (146, 147, 148). In the absence of Bcl11b, T cell development arrests at the DN2 stage, while precursors retain their ability to differentiate into NK cells.

Bcl11b is expressed in a proportion of ILCP that co-express ID2 or PLZF (Fig. 1), which exclusively generate ILC2 (145). Bcl11b is thought to promote ILC identity by simultaneously stabilizing expression of ILC2 lineage genes, while suppressing expression of ILC3 lineage genes (149) (Korchagina et al. 2023). Not surprisingly then, *Bcl11b*-deficient mice show impaired development and function of ILC2 and loss of ILC2 identity (140, 144, 145).

Human BLCB11 deficiency manifests with widely variable pathogenesis yet consistently disrupts normal development of both nervous and immune systems. All documented patients lacking functional BCL11B exhibit impaired T cell development and severely reduced ILC2 numbers in the periphery, despite varying degrees of immune dysfunction—ranging from LS (150) to autoimmunity (151) or even absence of overt pathology (110).

### Reconstitution of ILC following HSCT for the treatment of SCID

HSCT is a mainstream treatment option for SCID and CID patients, aiming to improve/restore immune competence by replacing the defective host immune system with a immunocompetent graft. The kinetics of immune reconstitution vary between different immune cell lineages, including ILC. NK cells, are among the earliest immune cells to reconstitute, typically restoring within 30–45 days after HSCT (152). This rapid reconstitution is facilitated by their development directly from hematopoietic precursors in the bone marrow. In contrast, T cells require 6 mo or longer to fully reconstitute due to their dependence on thymic maturation (153). ILC subsets (ILC1, ILC2, and ILC3) reconstitute much more slowly, often requiring 12 mo or more to recover (153). This delayed reconstitution may be attributed to the niche-specific requirements of these cells, which depend on tissue-specific signals for development and maintenance. Factors affecting ILC reconstitution include conditioning regimen, graft source (with cord blood potentially offering advantages for ILC recovery), and posttransplant inflammation or graft-versus-host disease (GvHD).

Residual host-derived hematopoietic cells can impede donor ILC engraftment, leading to partial recovery and potential immune dysfunction (152). Studies have shown that myeloablation facilitates robust ILC recovery, likely by providing a “clean slate” for donor-derived hematopoietic cells to repopulate the immune system (103, 152). For example, in patients with DOCK8 deficiency undergoing HSCT following myeloablative conditioning, all peripheral blood ILC subsets are successfully reconstituted. However, when myeloablation is not used, ILC reconstitution often remains incomplete (132).

Most studies assessing ILC recovery rely on peripheral blood samples, as tissue biopsies are often unavailable in posttransplant patients (152, 153). While peripheral blood analysis provides valuable insights, it may not fully capture the dynamics of tissue-resident ILC populations that are crucial for barrier immunity and tissue repair. Evidence suggests that delayed reconstitution of ILC contributes to posttransplant susceptibility to infection, particularly at mucosal barriers (152, 153). Moreover, activated ILC, particularly ILC2, have been associated with reduced susceptibility to GvHD; conversely, delayed or incomplete ILC recovery correlates with an increased risk of GvHD and poorer clinical outcomes, underscoring the importance of timely ILC reconstitution and confirming their protective role in regulating inflammation and maintaining tissue homeostasis (152, 153).

### IEI associated with A- or hypo-gammaglobulinemia

#### X-linked agammaglobulinemia (XLA)

XLA is a primary immunodeficiency characterized by significant reductions in B cells and immunoglobulins due to genetic mutation in the Bruton tyrosine kinase (*BTK*) gene. While XLA primarily affects B cells, limited BTK expression has been observed in ILC subsets in both murine models and human XLA patients (154).

Studies in XLA-mouse models indicate that NK cell development and function remain largely intact (155). Similarly in human XLA patients, NK cells are generally present in normal numbers, with functional analyses confirming that NK cell-mediated innate immunity is preserved. This may explain why XLA patients generally do not experience increased susceptibility to viral infections typically controlled by NK cells and T cells (156).

In addition, investigation of peripheral blood ILC subsets in XLA patients (*n* = 4), performed in the context of a larger CVID study, reported relatively normal levels of total ILC (lin-CD117^+^ or lin-CD117^−^) cells, with slightly elevated ILC2 (112). This finding has not yet been confirmed by other studies. Functional studies concerning helper ILC functions in response to IVIG treatment in XLA patients are also lacking.

#### Common variable immunodeficiency (CVID)

CVID is characterized by hypogammaglobulinemia and recurrent infections, with one third of CVID patients exhibiting additional immune dysregulation such as autoimmunity and chronic inflammation. Multiple studies have reported significant reductions in circulating ILC2 in CVID patients, particularly in those with secondary complications (112, 157). Further, Friedmann et al. discovered that ILC2 reduction in CVID occurs as part of a broader imbalance of ILC subsets, noting that ILC3 were enriched in the peripheral blood of CVID patients with immune dysregulation as compared to those only with infections (157). This finding was corroborated by Cols et al., who reported not only expansion of inflammatory ILC3 (IL-23R^+^, IFN-γ^+^IL-22^+^ phenotype) in peripheral blood but also in the intestinal biopsies of CVID patients with enteropathy in the inflamed but not normal tissue (158), suggesting that ILC3 may play a role in the autoimmune and intestinal complications in these patients.

In addition to numerical changes, CVID patients exhibit functional deficits—reduced cytokine production by ILC2 and increased activity in ILC1 and ILC3. Friedmann et al. and Geier et al. both reported impaired production of IL-5 and IL-13 by ILC2 in CVID, possibly contributing to defects in mucosal immunity and barrier repair and exacerbating susceptibility to infections (112, 157). Cols et al. showed that expanded ILC1-like cells produce elevated levels of IFN-γ and TNF-α (158). This proinflammatory cytokine skewing likely plays a role in driving chronic inflammation, enteropathy, and autoimmunity in CVID. The expansion of inflammatory ILC3/ILC1-like cells is closely associated with chronic inflammatory conditions, further establishing a link between ILC subset homeostasis and clinical pathogenesis (159). However, the precise mechanisms by which ILC3 contribute to mucosal and systemic immune dysregulation in CVID remain poorly defined. It is unclear whether the observed ILC3 expansion is a compensatory response or a driver of inflammation. Furthermore, the upstream signals promoting ILC3 differentiation and their interactions with microbiota and other immune cells in CVID remain unexplored. Future studies are needed to dissect the tissue-specific roles of ILC3, define their antigenic and cytokine responsiveness, and determine their potential as therapeutic targets in CVID-associated autoimmunity and enteropathy.

### IEI associated with cytoskeletal defects

#### DOCK8 deficiency

DOCK8 functions as a guanine nucleotide exchange factor that activates cell division control protein 42 (CDC42), a key cytoskeletal regulator (160, 161). This DOCK8-mediated CDC42 activation signaling is essential for normal signaling via both TCR and cytokine receptors. Disruption of this mechanism impairs development and function of multiple immune cell types, including T (Th17, Th2, and Treg), B, and NK cells, and ILC (160, 161, 162). DOCK8 plays a pivotal role in maintenance of ILC2 and ILC3 numbers, subset distribution, and cytokine production in both mice and humans (160, 161).

Multiple researchers studying various mouse models of Dock8 deficiency found reduced numbers of ILC3, mirrored by functional impairments in ILC3-mediated cytokine production (54, 163). Specifically, IL-23–mediated IL-22 production by ILC3 was impaired, suggesting DOCK8 may be regulating IL-23R/STAT3 axis, possibly accounting for the observed increase in infectious susceptibility by enteric pathogens (54). DOCK8-dependent ILC3 regulation also appears to involve survival mechanisms, where ILC3 were shown to undergo more apoptosis in the absence or dysfunction of DOCK8 (54). In contrast, studies of ILC2 numbers showed mixed results—some found no change (54), while others reported increased ILC2 accompanied by elevated levels of IL-5 and IL-13 in the intestines (164). However, in mice two studies have shown normal IL-7R expression, which may explain normal ILC2 levels.

The impaired cytokine responses in DOCK8-deficient ILC align with broader immune dysfunctions observed in DOCK8 deficiency, including recurrent infections and allergic disease (165, 166, 167). Notably, LOF mutations in DOCK8 lead to autosomal recessive hyper-IgE syndrome (HIES), a well-characterized autosomal recessive IEI. DOCK8-deficient patients exhibit reductions in peripheral ILC2 and ILC3 numbers, low IL-7Rα surface expression, and impaired IL-7–mediated STAT5 signaling (132). In both mice and humans, ILC3 were shown to undergo more apoptosis in the absence or dysfunction of DOCK8. As in mice, DOCK8 deficiency is associated with impaired survival of these ILC subsets, manifested in sorted ex vivo human ILC by less proliferation and more apoptosis (132). These ILC also displayed reduced numbers of IFN-γ, IL-17A, IL-22, and GM-CSF transcripts, collectively demonstrating the importance of DOCK8 in regulating ILC survival and function.

#### Wiskott–Aldrich syndrome (WAS)

WAS protein (WASP) is a hematopoietic-specific regulator of the actin cytoskeleton, playing a pivotal role in immune cell functions (168, 169). WASP LOF variants lead to combined immunodeficiency with syndromic features (IUIS 2024 classification, Table IIa), while gain-of-function (GOF) variants are associated with non-syndromic congenital defects in phagocytes and neutropenia (IUIS 2024 classification, Table Va) (102).

A recent study by Biswas et al. revealed a significant reduction in ILC3 numbers in the gastrointestinal tracts of WASP-deficient mice, resulting in impaired expression of antimicrobial peptides in response to IL-22, and increased susceptibility to *Citrobacter rodentium* infection (109, *Preprint*). Notably, the reduction in ILC3 was not observed in germ-free WASP-deficient mice, indicating that the presence of commensal microbiota influences this phenotype. The study also noted that ILC3-like cells were diminished in the gastrointestinal tracts of patients with WAS. These findings suggest that WASP plays a crucial role in ILC3 maintenance and function in a microbiota-dependent manner. This supports previous research on DOCK8 deficiency (54, 132), highlighting the broader importance of cytoskeleton proteins in the survival and function of adult ILC3 (109, *Preprint*).

#### Diaphanous-related formin 1 (DIAPH1) deficiency

DIAPH1, a formin family member widely expressed in mammals, functions as a linear actin nucleator (170, 171). Both mice and humans with DIAPH1 deficiency develop immunodeficiency, supporting the role of cytoskeletal proteins in immune function (172). Recent studies reveal that DIAPH1 LOF mutations result in major ILC defects (111), consistent with impairments observed in other cytoskeletal deficiencies like DOCK8 (54, 132) and WASP (109, *Preprint*).

Patients with DIAPH1 deficiency present with seizures, cortical blindness, microcephaly syndrome, and CID (172). In addition, DIAPH1-deficient patients exhibit significant deficiencies in NK cell cytotoxicity and cytokine production, as well as dramatic numerical deficits in helper ILC, implicating DIAPH1 in the development and function multiple ILC populations (111). This study also revealed impaired cytokine-mediated signaling for IL-7/STAT5, IL-2/STAT, and IL-15/STAT5 axes. Together, these findings illustrate the important role of DIAPH1 in the development and function of ILC, in addition to other immune cell types (173, 174). Further research is necessary to delineate the specific mechanisms involved.

#### Leukocyte adhesion deficiency I (LAD1), lymphocyte function-associated antigen-1 (LFA-1), and intercellular adhesion molecule (ICAM-1)

LAD1 is a rare disorder caused by mutations in β2 integrin family members and characterized by the inability of immune cells to properly migrate to sites of infection or inflammation (175). Patients harboring LOF mutations in LFA-1, a heterodimeric β2 integrin composed of CD11a and CD18, show elevated numbers of peripheral blood ILC3, but not ILC1 or ILC2 (107). This suggests LFA-1 deficiency creates an environment skewed toward type 3 immune responses. These findings are consistent with earlier reports of elevated IL-17 expression and increased Th17 activity in these patients (176).

LFA-1 and its ligand ICAM-1, are adhesion molecules crucial for the migration, localization, and function of immune cells, including ILC (107, 175). In mice, LFA-1 mediates ILC2 trafficking to the lungs during inflammatory responses to allergens like *Alternaria alternata* (177). Hurrell et al. confirmed this by showing impaired ILC2 lung infiltration and reduced airway inflammation triggered by IL-33 stimulation in LFA-1 KO mice; LFA-1 deficiency did not affect ILC homing to the lungs during homeostasis (178). Similarly, ICAM-1 deficiency leads to attenuated allergic inflammation and reduced ILC2 accumulation in the lungs, but not to a lesser degree compared to LFA-1 deficiency (178). ICAM-1^−/−^ mice exhibit significant reductions in ILC2 numbers in bone marrow and peripheral tissues (179). Bone marrow chimera experiments show that ICAM-1 intrinsically regulates ILC2 development and cytokine production through ERK-mediated regulation of GATA3. In addition, LFA-1/ICAM-1 interactions mediate ILC2 crosstalk with stromal cells, especially in adipose tissue, supporting ILC2 proliferation and activation (180). ILC2-derived cytokines, IL-4 and IL-13, induce eotaxin production, facilitating eosinophil recruitment and maintaining adipose tissue homeostasis (180). Further research is needed to translate these findings to human ILC2 or other ILC subsets.

### IEI associated with TF defects

#### RORγt deficiency

RORγt, encoded by its gene *RORC*, is nuclear receptor TF, which works as a master regulator for both Th17 cells and ILC3 (181). The impact of *RORC* defects on ILC development and function have been studied in mice and humans in the context of ILC3 subsets, LTi cells, and natural cytotoxicity receptor (NCR)^+/−^ ILC.

Studies on *Rorc*^−/−^ mice demonstrated the complete absence of ILC3 subsets, including LTi cells and postnatal ILC3 (NCR^+^ and NCR^−^ subsets). This results in failed development of lymph nodes and Peyer’s patches (4, 182, 183). Rorc^−/−^ mice also showed reduced production of IL-17 and IL-22, cytokines crucial for maintaining mucosal immunity and responding to bacterial infections. Pharmacological inhibition of RORγt via select inhibitors specifically spared ILC3 function by decreasing IL-17 and IL-22 production and differentiation of Th17 cells, while leaving other ILC and T cells subsets intact (184, 185, 186). These results confirm findings from *Rorc*-deficient mice and highlight RORγ as a potential therapeutic target for autoimmune and inflammatory diseases (187).

In humans, *RORC* mutations lead to a complete lack of ILC3 subsets, including LTi cells, and impaired lymphoid organogenesis (105). Patients with *RORC* deficiency exhibited severe deficiencies in secondary lymphoid structures, such as lymph nodes and Peyer’s patches, consistent with lack of LTi cells. These deficiencies contribute to impaired mucosal immunity and increased susceptibility to fungal and intracellular bacterial infections (105). Nonetheless, patients lack global features of immune dysregulation and autoimmunity (105). Studies of *RORC*-deficient human IEI patients, and comparable mouse models, underscore how *RORC* governs ILC biology, providing insight into its influence on immune regulation and homeostasis.

#### GATA3 deficiency

GATA3 is a zinc finger TF initially defined as the master regulator of Th2 differentiation (188) but subsequently shown to also be essential for the development and function IL-7Rα–expressing ILC (189). Like Th2, GATA3 is especially important for the maintenance of ILC2 (190), regulating the expression of genes responsible for ILC2 identity and function, like Il5, Il13, Il1rl1, Il2ra, Il9r, and Ccr8 (189).

GATA3-deficient mice show decreased numbers of ILC2 across tissues—bone marrow, lung, liver, and small intestine (190, 191). Additionally, GATA3 shapes the development of Lti cells and NKp46^+^ ILC3 by modulating the balance between T-box TF TBX21 (T-bet) and RORγt TF. Beyond these developmental roles, GATA3 maintains homeostasis via regulation of IL-7Rα expression.

GATA3 deficiency in human patients causes a rare autosomal dominant disorder variably called HDR or Barakat syndrome, characterized by hypoparathyroidism, sensorineural deafness, and renal dysplasia (192). Although GATA3 has been established as a critical regulator of ILC development and function in mice, particularly for ILC2, human studies examining ILC subsets in GATA3-deficient patients remain limited/unknown. Further research in this area could provide valuable insights into the role of GATA3 in human ILC biology.

#### T-BET deficiency

T-bet is a master regulator for Th1 cell differentiation. It orchestrates the Th1 program by binding to the promoter of *IFNG* and turning on other Th1-associated genes (193, 194). Beyond Th1 cells, T-bet is also essential for the proper functioning of NK and CD8^+^ T cells, influencing their cytotoxic activity and cytokine production (195).

In the context of ILC, T-bet plays a pivotal role in the development and function of various subsets. ILC1 are heavily reliant on T-bet for their differentiation and maintenance, whereas NK cells depend more on eomesodermin (44, 65, 196, 197, 198). Notably, T-bet deficiency leads to a significant reduction in ILC1 populations, particularly in the liver and intestine, underscoring its crucial role in ILC1 biology (44, 196, 197).

T-bet is also instrumental in ILC plasticity (72, 74). Under certain conditions, such as exposure to specific cytokines, ILC3 can transdifferentiate into ILC1—a process that requires T-bet. In the absence of T-bet, this ILC3-to-ILC1 transition is impaired, leading to an accumulation of ILC3 even in inflammatory settings (72, 74).

Furthermore, the development of NCR^+^ ILC3 is dependent on T-bet. This subset emerges postnatally upon exposure to microbiota and food-derived antigens, highlighting T-bet’s role in adapting the immune system to environmental cues encountered after birth (198).

In humans, TBX21 deficiency has been identified as an IEI (OMIM# 619630). This IEI leads to increased susceptibility to mycobacterial infections and type I immunity due to impaired development and function of various lymphocyte subsets, including cytotoxic ILC1 subset NK cells and innate-like adaptive lymphocytes (iNKT, mucosal-associated invariant T [MAIT], and Vδ2^+^ γδ T cells) (199). However, the specific impact of T-bet deficiency on human helper ILC1 and ILC3 subsets remains unclear; studies in humanized mouse models are needed.

### IEI associated with cytokine receptor defects

IL-23R, a heterodimeric cytokine receptor composed of IL-12Rβ1 and IL-23Rα subunits, plays a role in both innate and adaptive immunity. It is expressed on NK and ILC3, as well as Th17, γδ T, MAIT, and a subset of NKT cells. ILC3 are activated upon exposure to IL-23 (15) and IL-23R activation triggers a variety of signaling cascades, primarily through the JAK–STAT pathway (200, 201, 202). Similarly, the IL-12 receptor (IL-12R) is composed of IL-12Rβ1 heterodimerized with IL-12Rβ2. IL-12R is expressed on ILC1 and NK cells, and its activation is necessary for IFN-γ production by these cells (200, 201, 202).

Murine studies have unequivocally established an essential role for IL-23R–mediated signaling in ILC3 differentiation (203), expansion, and effector functions (85, 87, 204, 205). In these studies, IL-23R deficiency dramatically reduced ILC3 cell numbers, increasing intestinal pathology and limiting ILC3-associated protective immune responses.

Monogenic deficiencies of IL-23R, IL-12Rβ1, and IL-12Rβ2 are listed as “defects in intrinsic and innate immunity” in the 2024 IUIS IEI classification matrix (Table 6) (102). Martínez-Barricarte et al. found that patients with these deficiencies maintained normal peripheral total ILC counts, though their study lacked detailed peripheral subset identification and tissue-resident ILC information (106). These and other studies revealed that IL-23R deficiency leads to mycobacterial susceptibility but, unlike deficiencies in IL17F, IL17RA, or ACT1, does not consistently cause fungal susceptibility or chronic mucocutaneous candidiasis. This suggests that redundant mechanisms within type 3 immunity may compensate when IL-23R is defective. It remains unclear whether this redundancy comes from RORγt^+^ innate cells such as ILC3 or other lymphoid populations like γδ T, MAIT, and NKT subsets.

Due to the difficulty in obtaining patient tissue samples and the rarity of human IL-23R, IL-12Rβ1, and IL-12Rβ2 deficiencies, it is currently unclear how defects in the IL-23/IL-23R axis impact human ILC3 generation, expansion, and function.

### IEI associated with STAT signaling defects

#### STAT3 and ILC

The TF STAT3 regulates ILC3 survival and development when activated by specific cytokines (IL-6, IL-21, and IL-23) or growth factor receptors (EGFR, Flt3, and c-Kit). Notably, IL-23 not only activates STAT3 but is also essential for the expansion of ILC3 and their production of IL-22, a cytokine that helps maintain gut homeostasis and protects against inflammation (43, 206, 207, 208).

Because germline KOs of STAT3 are lethal in mice, the effect of STAT3 deletion has only been examined in conditional KO systems. Specific deletion of STAT3 in Rorc^+^ cells does not impact ILC3 numbers but restricts their ability to produce IL-22, showing that although STAT3 is not required for murine ILC3 ontogeny, it still affects the ability to defend against enteral pathogens (209). Conditional deletion of STAT3 in NCR+ ILC3 also led to reduced IL-22 production (52). In contrast, a decrease in ILC2 and ILC3 number, but not function, is observed Flt3l^−/−^ mice, indicating that STAT3 may be required for development of ILC2/3 (129). These findings—STAT3 is vital for the expansion and function of murine ILC3—were confirmed by our studies conducted in IL-23R KO mice (87).

The role of STAT3 in human ILC3 development and function has not yet been directly demonstrated. Studies have shown that IL-23 activation is important in maintaining ILC3 identity, and in the trans differentiation of ILC2 to ILC3 (210). In addition, Lim and colleagues (63) report that c-Kit (CD117)+ ILC progenitors can differentiate into ILC3 when stimulated with IL-23. These studies, however, did not specifically investigate the role of STAT3 in this process.

Patients with LOF mutations in STAT3 develop autosomal dominant HIES, characterized by elevated serum IgE levels, recurrent infections, and eczema (211, 212). These individuals could be expected to have impaired function of ILC3, leading to reduced IL-22 production and increased susceptibility to mucocutaneous infections. Our recent study showed no change in ILC numbers present in peripheral blood; however, further studies are needed to characterize tissue-resident ILC3 populations (132). STAT3 GOF mutations manifest as early-onset autoimmunity often accompanied by recurrent infections, particularly in the lungs (213). Such STAT3 mutations could lead to dysregulated ILC activity, contributing to tissue inflammation and autoimmunity, but this connection has not yet been investigated. Furthermore, the specific effects of both STAT3 LOF and GOF mutations on human ILC subsets, their function, and plasticity require further study.

#### STAT4

STAT4 is important for IL-12R signaling and is also activated by IL-23R via its subunit IL-12Rβ1. IL-12R is expressed primarily by NK and ILC1 (214, 215, 216, 217). Murine studies reveal that neither NCR-specific deletion nor germline deletion of STAT4 alter the homeostatic pool of intestinal or other tissue (spleen, liver, and bone marrow)-resident ILC (198, 218, 219). However, in models of IBD, STAT4 deletion led to significant reduction in IFN-γ production by NK and ILC1 (215). Human STAT4 autosomal recessive mutations are associated with disabling pansclerotic morphea (220). These mutations are listed as autoinflammatory disorders in the 2024 IUIS IEI classification matrix, Table 7 (102). ILC have not been yet studied in human patients with STAT4 pathogenic variants.

#### STAT5

STAT5 is activated by various cytokine receptors, primarily IL-2, IL-7, IL-15, GM-CSF, IL-3, IL-5, IL-4, and IL-9 (221). IL-23R signaling has also been shown to activate STAT5, although the consequence of such activation remains unclear (222). STAT5 plays critical roles in all ILC subsets, especially in development and function via integration of signals from IL-7 (103, 223, 224). Additionally, STAT5 processes signals from IL-15, which is particularly important for ILC1 and ILC2 development (128, 130). A variety of mutations in human STAT5 have been documented and are associated with chronic inflammatory diseases (Crohn’s and AD), as well as multiple forms of cancer (225). ILC have not been studied in these patients.

### Crosstalk between ILC and other immune and stromal cells in IEI

ILC do not act in isolation but instead engage in bidirectional crosstalk with a broad array of immune and nonimmune cells to regulate tissue immunity and homeostasis (226, 227, 228). In the context of IEI, defects in ILC development or function can result in profound non-cell-autonomous consequences, amplifying immune dysregulation beyond the primary molecular lesion.

The most vital interactions occur between stromal and organizer LTi cells at the lymphoid anlagen during lymphoid organogenesis (229). These interactions involve both soluble factors and their corresponding receptors, e.g., lymphotoxins, chemokines, and cytokines (especially Il-7/IL-7R). ILC also interface extensively with epithelia. ILC2 and ILC3 respond to epithelial-derived alarmins such as IL-25, IL-33, and TSLP, and in turn secrete amphiregulin, IL-22, and IL-13 to promote epithelial regeneration and antimicrobial defense (66, 99). In IEI with actin cytoskeletal defects, such as WASP, CARMIL2, DIAPH1 and DOCK8 deficiency, impaired ILC responses to alarmins may underlie defective barrier immunity and microbial translocation (109, 132, *Preprint*, 111, 230). Notably, epithelial damage and altered ILC-epithelial feedback may also promote inappropriate immune activation and advance tissue pathology, creating a self-amplifying cycle of dysregulation.

Another key axis of interaction involves ILC and dendritic cells (DC). Such interactions can be direct, taking the form of immunological synapses through receptor-ligand pairs such as LTRβ/LTαβ and DNAX accessory molecule 1/CD112 or CD155. Alternatively, ILC–DC interactions can be indirect, mediated by soluble molecules, including cytokines and chemokines. Examples include IL-23, IL-33, TSLP, IL-1β produced by DC, and GM-CSF and chemokines produced by ILC (231).

ILC also interact with cells of the adaptive immune system (23). ILC3 express MHC class II and can present antigens to CD4^+^ T cells, thereby influencing adaptive immunity (27, 30). This interaction has been shown to promote regulatory T cell differentiation and to limit Th cell responses in the gut, maintaining immune tolerance to commensals. In IEI, such as WAS and DOCK8 deficiency, where ILC3 numbers/function are compromised (109, 132, *Preprint*), these regulatory loops may be disrupted, contributing to chronic inflammation and mucosal barrier defects. The interaction between ILC and adaptive lymphocytes adds another layer of complexity that may be affected in IEI. ILC2 can shape CD4^+^ T cell polarization through cytokine secretion, while ILC3 influence the balance between regulatory and effector T cells via IL-22 and MHCII-dependent antigen presentation mechanisms (232). In RAG-deficient patients and mouse models that lack T cells, the unopposed activity of ILC may lead to pathological inflammation (62, 104), further underscoring the need for balanced interplay. Conversely, in disorders such as STAT3 or IL2RG deficiency, shared signaling defects may affect both ILC and T cells, compounding immune dysfunction (233, 234).

Together, these observations emphasize that ILC dysfunction in IEI not only impairs innate immunity but also disrupts a network of intercellular communication crucial for immune regulation. Future work dissecting ILC-driven crosstalk in tissue niches—using single-cell approaches and spatial transcriptomics—may reveal new therapeutic targets to restore immune homeostasis in IEI.

### Environmental and epigenetic modulation of ILC in IEI

ILC are highly sensitive to extrinsic environmental cues, which shape their differentiation, function, and tissue distribution. In the context of IEI, where intrinsic genetic lesions impair immune regulation, these extrinsic factors may exacerbate or modulate disease phenotypes, contributing to the observed inter-individual heterogeneity. Understanding how microbiota, infection, inflammation, and epigenetic mechanisms influence ILC is essential to contextualize ILC dysfunction in IEI.

Microbiota and mucosal colonization are well-established regulators of ILC homeostasis (235). Commensal-derived signals promote the expansion and activation of ILC3, particularly in the intestine, through the engagement of pattern recognition receptors on epithelial and myeloid cells that produce IL-1β and IL-23 (72, 85, 236, 237, 238). In IEI that affect the microbiome (239), the dysbiosis-induced dysregulation of ILC3/ILC1 could lead to early-IBD and barrier defects.

Infectious triggers may also profoundly modulate ILC phenotypes. Viral infections, for example, may induce the conversion of ILC3 to IFN-γ–producing ex-ILC3 in a T-bet–dependent manner (74). This plasticity may be beneficial in some contexts but pathogenic in others, such as IEI with defective viral clearance, e.g., GATA2 and MCM4 deficiencies, where chronic stimulation may drive skewed ILC polarization and inflammation (5).

Inflammatory cytokine milieus, including persistent elevation of IL-18, IL-1β, and TGF-β, are potent drivers of ILC conversion. For example, exposure to TGF-β has been shown to reprogram NK cells and ILC1 toward a noncytotoxic phenotype (240). In patients with IEI involving aberrant cytokine regulation, such as STAT1 GOF, CYBB, and WAS, these inflammatory circuits may also skew ILC phenotypes, diminish anti-pathogen immunity, and promote chronic inflammation.

Epigenetic mechanisms further modulate ILC function and fate decisions. Histone modifications and changes in chromatin accessibility dynamically influence ILC subset identity and responsiveness to external cues (241). More investigation is needed to understand how mutations in epigenetic regulators, like ARID1B, DNMT3B, and CHD7, may affect ILC development, plasticity, or function in human IEI.

Lastly, food-derived molecules can significantly impact ILC subsets and cytokine profiles, thereby altering protective immune responses and influencing intestinal and other inflammatory disease outcomes. Such molecules include aryl hydrocarbon receptor agonists (51, 77, 242, 243, 244), fatty acids (245), and vitamins A (246, 247) and D (102, 248, 249).

Taken together, these insights support a model where gene–environment interactions and epigenetic landscapes jointly regulate ILC behavior in IEI. Future studies integrating single-cell transcriptomics and epigenomics with tissue microbiome and cytokine profiling in patient samples will be essential to decipher the extrinsic modulation of ILC in IEI and their role in disease heterogeneity.

## ACT 3: Understudied and unrecognized genius—do ILC represent pleonasm or neologism?

In recent years, ILC have emerged as important components of immune responses in immunocompromised mice (12, 74, 246, 250, 251, 252, 253), yet their necessity remains debated in immunocompetent hosts. While they contribute to protective immunity in various conditions and resemble helper T cells in many ways, the question persists—are ILC merely redundant cells whose absence can be compensated by other immune elements or essential components with unique functions? This section examines evidence from both perspectives, evaluating studies in mouse models and human subjects that argue for either redundancy or irreplaceability of these cells.

Studies suggesting redundancy have shown that in immunocompetent mice, components of adaptive immunity can compensate for ILC defects against certain pathogens (52, 55, 254, 255). These investigations had notable limitations, in that ILC targeting relied on lineage markers shared with other immune cells, e.g., NCR^+^ and CCR6^+^, or failed to fully deplete all ILC subsets. In humans, Vély et al. conducted a landmark study investigating ILC necessity in antimicrobial immunity by examining pediatric and adult SCID patients before and after HSCT (103). The study revealed that patients who did not receive myeloablation prior to transplantation showed poor ILC reconstitution in both peripheral blood and tissues, particularly in SCID patients with *JAK3* and *IL2RG* deficiencies. In contrast, myeloablation enabled restoration of both peripheral and tissue ILC populations. Importantly, long-term follow-up of SCID patients with persistent ILC deficiency after HSCT showed no increased susceptibility to infection despite ongoing ILC-penia, suggesting compensatory mechanisms by other immune components. Despite the limited number of tissue biopsies, this study provides compelling evidence for immune redundancy in the absence of ILC in humans (103).

Conversely, recent studies in mice using more selective ILC depletion methods have revealed unique and nonredundant roles for these cells (256, 257, 258, 259). Moreover, additional studies examining immunocompromised mice provide overwhelming evidence that ILC1, ILC2, and ILC3 are critical to protective immunity against type I, type 2, and type 3 pathogens (54, 55, 56, 77, 85, 256, 257, 258). In humans, other post-HSC transplant studies have shown that reduced numbers of ILC, particularly CD69^+^ ILC, are associated with acute GvHD (152), while ILCP, particularly NKp44^+^, CD25^+^, and CCR6^+^ ILCP, are associated with chronic GvHD (153). As discussed in the Reconstitution of ILC following HSCT for the treatment of SCID section of this review, ILC are successfully restored in patients post-HSCT, provided they received pretransplant myeloablation (43, 132, 260). These findings support the notion that human ILC may have irreplaceable roles in a context-dependent manner.

The functional redundancy observed in some studies likely depends on multiple factors, including microbiome composition, environmental exposures, genetic modifiers, and residual adaptive immune activity. With the ongoing discovery of more specific ILC markers and development of precise biochemical and genetic tools, our understanding of these cells’ unique functions will continue to expand. This advancing knowledge will help resolve the current debate regarding whether ILC are merely redundant players, constituting a pleonasm in host defense, or represent a terribly “understudied and unrecognized genius”—a neoplasm in our framework of immune host defense and self-tolerance.

## ACT 4: The plot thickens—outstanding questions, challenges, and future directions for the use of the “human model” to uncover novel ILC-directed therapies in IEI and beyond

Despite their emerging prominence in immunity, ILC remain underappreciated as therapeutic targets. Much like Christine Daaé stepping into the limelight, these cells have long been overshadowed by their more famous T cell counterparts, with only NK cell–based therapies making their way onto the clinical stage, while other ILC subsets remain largely unexplored. Although their indispensability in protective immunity and homeostasis remains debated, ILC appear to be significant players in the orchestration of mucosal immunity, tissue repair, and immunopathology. Despite progress in defining their roles, major questions remain. What are the precise signals that govern their lineage commitment and plasticity in human tissues? How do ILC integrate metabolic, cytokine, and stromal cues to mediate context-specific responses—protective or pathogenic? Can ILC develop memory-like functions, and if so, can these be therapeutically harnessed?

Human IEI present a unique opportunity to dissect the genetic and molecular pathways governing ILC biology, yet remain an understudied avenue in comparison to their adaptive immune counterparts. Most human studies rely on circulating ILC or in vitro culture models, which, much like the Phantom’s fleeting appearances, offer only glimpses of their in vivo function. In the context of IEI patients, small cohort sizes and limited access to tissue biopsies constrain progress. To overcome these barriers, humanized mouse models, organoid systems, and advanced gene-editing approaches, including CRISPR/Cas9 and conditional KOs, will be essential. Ultimately, a true human model will only be achieved through coordinated, cross/interdisciplinary, international collaboration that enables pooling of patient data, generalization of findings, and movement of ILC research from the shadows into the spotlight of definitive immunological understanding.

To fully define the roles of ILC in human health and disease, particularly in IEI, a sharper toolkit is needed. More refined genetic and pharmacological approaches are needed to precisely target specific ILC subsets without affecting other cell populations. These include novel Cre mouse lines that selectively target ILC2 and ILC3 subsets (250, 252, 253) and subset-specific inhibitors like RORγt inhibitors for ILC3. Such tools will enable more precise research and therapeutic applications while revealing the nonredundant functions of ILC without confounding contributions from other cell types. Spatial and single-cell approaches to capture the complexity of tissue-resident ILC in situ are also imperative in human studies where mechanistic studies are inherently difficult to design/execute. Beyond technological innovation, progress will depend on deep clinical phenotyping, tissue access, and scalable collaborations that connect rare IEI cases to mechanistic insights.

Therapeutically, helper ILC remain an underexplored frontier. Unlocking their potential—whether through small-molecule inhibitors, cytokine modulation, or ILC-based cell therapies—will require rigorous target validation in humanized mouse models and clinical trials. The translational path forward lies in identifying nonredundant, disease-driving functions of ILC subsets and integrating this knowledge into personalized, precision immunotherapy platforms.

The curtain is rising on a new act in immunology—one where ILC move from enigmatic understudies to lead actors in both fundamental biology and translational medicine. The challenge now is to illuminate the hidden choreography of these innate players and write the next act with clarity, rigor, and imagination.
